# Above- and below-ground biomass relationships of *Leucaena leucocephala* (Lam.) de Wit in different plant stands

**DOI:** 10.1371/journal.pone.0207059

**Published:** 2018-11-15

**Authors:** FangYan Liu, ChengJie Gao, Min Chen, Kun Li

**Affiliations:** 1 Research Institute of Resources Insects, the Chinese Academy of Forestry, Kunming, Yunnan Province, China; 2 Desert Ecosystem Research Station in Yuanmou County of Yunnan Province, State Forestry Administration of China, Yuanmou, Yunnan Province, China; 3 College of Life Science, Southwest Forestry University, Kunming, Yunnan Province, China; Estacion Experimental del Zaidin, SPAIN

## Abstract

The above and below–ground biomass (AGB and BGB) relationship is often used to assess the impact of biotic and abiotic effects on the growth and development of individual plants. The AGB and BGB relationship of the same tree species in different habitats can change significantly because of environmental stress. To investigate how the tree size, the biomass allocation and BGB/AGB ratio of *Leucaena leucocephala* (Lam.) de Wit varied according to spacing and mixed plant patterns in a valley–type savanna of southwest China, we examined the growth of *L*. *leucocephala*, and sampled 23 individuals for biomass measurement in each of four treatments (close/wide spacing of *Leucaena leucocephala* monocultures, mixed plantation of *Leucaena leucocephala* and *Eucalyptus camaldulensis*, and mixed plantation of *Leucaena leucocephala* and *Eucalyptus citriodora*), and then determined the regression relationships between AGB and BGB of *L*. *leucocephala* in different plant stands. Our results indicated that mixed planting significantly reduced all growth metrics for the tree sizes of *L*. *leucocephala* and increased the value of BGB/AGB. Changing plant spacing in monocultures had a significant impact on AGB and TB (Total Biomass) of *L*. *leucocephala*, but it had no significant effect on the other metrics. Within mixed plant schemes, *L*. *leucocephala* significantly reduced the biomass allocation to leaves and small roots and increased the allocation to coarse root biomass. There were no significant differences in tree size and biomass allocation of *L*. *leucocephala* between different spacing regimes in monocultures or between different mixtures in mixed plant stands. The correlation between BGB and AGB of *L*. *leucocephala* in all plant stands was consistent with the model of allometric growth, and AGB can be used to accurately estimate BGB. Interestingly, the correlations were not exactly the same. BGB and AGB in monoculture showed isometric growth, and their values in mixed plant stands showed allometric growth. BGB also increased faster than AGB. The findings indicated that *L*. *leucocephala* allocated more biomass to the root system when it was planted with *Eucalyptus*.

## Introduction

Biomass distribution in plants occurs as a result of environmental adaptation, and it plays a key role in supplying ecosystem services such as those related to primary production, carbon storage, soil conservation, and nutrient cycling [1–4]. In addition to such practical applications, knowledge about the biomass distribution is essential for understanding basic ecophysiological processes, especially those related to plant life history strategies and tree water balance [5–6]. Above–and belowground biomass (AGB and BGB) distribution is important for the study of plant biomass distribution strategies, often reflecting the requirements and competitiveness of plants with regards to soil water, nutrients, or light [7–10]. The ratio of below–and aboveground biomass (BGB/AGB) is a key index in plant ecology, and it is often used to assess the impact of biotic and abiotic effects on the growth and development of individual plants. Due to adaption to environmental stress, BGB/AGB ratio of the same tree species in different habitats typically changes significantly. This reflects the plasticity of plants. For example, the biomass distribution favors the stem when photosynthesis is limited, whereas the biomass is favorably distributed to the root when the absorption of mineral elements and water is limited [11–12].

Many studies have investigated the size and distribution of AGB and BGB in herbaceous plants [13–17]. However, there are relatively fewer studies on trees because obtaining the biomass of tree species is more difficult, especially with the limited available methods to sample roots [18–19]. To more accurately understand the biomass distribution characteristics of trees under diverse environmental conditions, more precise data are required to support the assessment.

Growth of nitrogen fixing plants plays a crucial role in the long–term development of artificial forests, especially in eucalypt forests [6, 20–23]. The commercial value of a eucalypt harvest may depend strongly on the size of individual trees, so the effects of nitrogen fixing plants on *Eucalyptus* are often emphasized. Studies have shown that mixed planting of nitrogen fixing trees and *Eucalyptus* can promote the growth of *Eucalyptus* [5, 22, 24]. Few studies have investigated how nitrogen fixing trees are affected by mixed planting with non–nitrogen fixing trees. Viera et al. [25] suggested that inclusion of nitrogen fixing trees in plantations of non–nitrogen fixing trees could increase the AGB of the nitrogen fixing trees. For instance, the inclusion of *Acacia mearnsii* in *Eucalyptus globules* plantations may increase the AGB of *A*. *mearnsii* [20–21, 23]. This effect has also been demonstrated in *Eucalyptus saligna* and *Albizia falcataria* [26], *E*. *saligna* and *Falcataria moluccana* [22], *Eucalyptus grandis* and *Acacia mangium* [5]. To our knowledge, very few studies have tested AGB and BGB allocation of nitrogen fixing trees in mixed plantations with non–nitrogen fixing trees and variable plant spacing.

*Leucaena leucocephala* (Lam.) de Wit is a nitrogen fixing plant native to tropical America. It is now widely distributed in the valley–type savanna of Jinsha River in China as an extremely important management option for ecological recovery in a region that experiences high risk of vegetation loss and soil erosion [27–28]. In the present study, we investigated the effects of plant spacing and mixed planting with non–nitrogen fixing trees (*Eucalyptus camaldulensis* and *E*. *citriodora*) on AGB and BGB allocation of *L*. *leucocephala* in the valley–type savanna of the Jinsha River. The main objectives of this study are (a) to understand how the biomass allocation and BGB/AGB ratio vary according to spacing and mixed planting patterns and (b) to determine whether the relationships between AGB and BGB of *L*. *leucocephala* in different plant stands of the valley–type savanna were consistent with the allometric growth models.

## Materials and methods

### Ethics statement

This work was approved by Desert Ecosystem Station in Yuanmou County of Yunnan Province, State Forestry Administration of China. No protected species were sampled.

### Site description and trial establishment

The study was carried out in the artificial vegetation restoration area of Desert Ecosystem Station in Yuanmou County of Yunnan Province, State Forestry Administration of China (25° 40' N, 101° 52' E), and the study area was approximately 30 km south of the Jinsha River. The site was situated in a relatively low elevation valley–type savanna (approximately 1, 200 m a.s.l.), where it was extremely hot and dry due to the influence of the monsoon, mountainous terrain, and canyons. The year is divided into a wet and dry season. Average annual rainfall (1991–2015) is 584 mm. The largest rainfall year (891 mm) occurred in 1998, and the driest year (471 mm) occurred in 2010. Rainfall distribution tends to be wet season dominant (June–October) with only 8% of the annual rainfall falling in the dry season (November–May of the following year) when evaporative demand is highest. The average annual potential evapotranspiration (1991–2015) is 3, 848 mm. Thus, the trees grow in an environment where the atmospheric water demand is on average 6.6 times the rainfall. The long–term average annual temperature is 22°C. The lowest average monthly temperature was 15°C (December), with an extremely low temperature of -1°C. The highest average monthly temperature was 27°C (May), with an extremely high temperature of 42°C. The annual accumulated temperature (≥10°C) was 8, 003°C (The meteorological data in this section were from the weather station of Yuanmou County). The main type of soils is classified as Ferralic Arenosols according to the FAO Taxonomy [29–30]. There is shallow topsoil, and the subsoil layers are deep, compact, and have a high level of gravel (mass fraction > 35%) with poor water retention. Thus, the trees grow in an environment where there is a high degree of desertification and severe soil erosion, with ravines on the soil surface.

The experimental forests were planted in 1998. In this paper, the experimental design consisted of four treatments (two spacing and two mixed plant treatments). The spacing treatments included 100% *L*. *leucocephala* at close spacing (1.5 m×3.0 m) and 100% *L*. *leucocephala* at wide spacing (3.0 m×3.0 m). Mixed plant treatments included 50% *L*. *leucocephala* + 50% *E*. *camaldulensis* at 3.0 m×3.0 m spacing which we will refer to as “M. W. *camaldulensis*” and 50% *L*. *leucocephala* + 50% *E*. *citriodora* at 3.0 m×3.0 m spacing which we will refer to as “M. W. *citriodora*” The average diameters at breast height (DBH) of *E*. *camaldulensis* and *E*. *citriodora* were 19.8±1.9 cm (Mean±SE) and 17.4±0.9 cm, the average heights were 11.2±1.0 m and 13.6±0.9 m, and the average crown diameters were 5.1±0.2 m and 4.7±0.1 m, respectively. We also used the parameters of average DBH, average height, and average crown diameter to describe the effect of the plant stand on growth of *L*. *leucocephala*.

### Biomass sampling

Sampling for biomass of *L*. *leucocephala* was carried out in the winter of 2015 when the trees were 17 years old. As a preliminary step, we randomly selected 100 trees in each of the four treatments and collected the data of each tree including the diameter at breast height (DBH), the height, and the crown diameter. The crown diameter was calculated according to the crown’s orthogonal projection on the ground. All of these data were used to verify the growth characteristics and the DBH class distribution of the plants in the four treatments. According to the DBH class distribution of the trees, 23 trees with different DBH in each of the four treatments were selected to sample biomass. For example, the DBH range of *L*. *leucocephala* in close spacing treatment ranged from 6.9 to 15.7 cm, and the DBH could be divided into 5 class levels. These classes included 6 cm<DBH≤8 cm, 8 cm<DBH≤10 cm, 10 cm<DBH≤12 cm, 12 cm<DBH≤14 cm, and 14 cm<DBH≤16 cm, and 4–5 trees were selected from each DBH class level.

### Above–ground biomass sampling

Each sample tree of *L*. *leucocephala* was cut at ground level (leaving 10 cm of stump) and the crown (including branches and foliage) was removed from the stem. The aboveground biomass (AGB) was separated into stem biomass, branch biomass, and foliage biomass. The stem at the top was cut at 3 cm diameter and the part smaller than 3 cm was included in the branches portion. Foliage was removed from the branches by hand, and both were weighed fresh in the field using a field dynamometer and the values were recorded. Subsamples of branches and foliage were labeled, bagged, and then transported to the laboratory of Desert Ecosystem Research Station in Yuanmou County of Yunnan Province, State Forestry Administration. The subsamples were then dried at 85°C to a constant weight for determination of water content. The dry weight for branches and foliage was then calculated. The stem was divided into the base (ground to 1.3 m), middle (1.3 m to halfway from top), and top. Diameters, lengths, and fresh weight were recorded for each stem section. Disks from the stem were taken at ground level, breast height, and top. Fresh weight was determined for each component and all samples were oven dried to a constant weight at 85°C. Weights were recorded to calculate dry weights of tree parts.

### Below–ground biomass sampling

Belowground biomass of *L*. *leucocephala* was determined by the stratified excavation method. For each tree selected for sampling, the scope of the root system excavation was set as a circle with a radius of 1.0 m and the base of the trunk as the center. The sample tree roots were harvested to a depth of 100 cm by manual excavation to collect total belowground biomass, and the root samples were weighed for each 20 cm of depth. Thus, the vertical distribution of below–ground biomass was divided into 5 layers, i. e., 0–20, 20–40, 40–60, 60–80, 80–100 (unit: cm). Due to the time involved in sampling and the much smaller number of roots encountered, excavation of the 50–100 cm depth was reduced to half of the area used for the 0–50 cm excavation. All roots from these areas regardless of their excavated source were included in the measurement of root biomass. Thus, the part of the root system of the sample tree that grew outside of the sampled area was not sampled, but any roots that grew into the soil from other trees were included. In any event, it was impossible to excavate and recover the entire root system for each sample tree because of root intermingling, the enormous effort required for excavation, root separation, and the destruction of plots that occurred during the excavation. It should be pointed out that the belowground biomass might not be sampled fully (in a 1 m radius around trees); and the BGB as well as BGB/AGB were likely underestimated.

The belowground biomass (BGB) was separated into the vertical taproot (the primary root) and the lateral root. The vertical taproot was excavated to a diameter (or diameters in cases where it split) of 2 cm, and the part smaller than 2 cm was included as the lateral root. According to the size of root diameter (RD), the lateral root was separated into small root (RD ≤ 1.0 cm), medium root (1.0 cm < RD ≤ 2.0 cm), and coarse root (RD > 2.0 cm). All root systems were extracted from the soil and separated from the soil particles and sediments by gentle shaking and removal by hand. The fresh weight was measured in the field. The root samples were oven dried to constant weight at 85°C and then used to calculate total oven–dry weights of root system parts.

### Data processing and statistical analyses

We analyzed the spatial distribution of vertical belowground biomass according to the root system distribution model (RSDM) proposed by Gale [31]:

$$Y=1-\beta^{d}$$

where, *Y* is the ratio of the sum of biomass from the topsoil (0 cm) to the depth *d* (cm) of the overall belowground biomass and *β* is the extinction coefficient. A larger *β* value indicates a greater proportion of deep root biomass; while a smaller *β*–value indicates that a greater proportion of the root system is present in the surface soil or in the lower level of the crown.

The regression between above–and below–ground biomass was developed using the equation: y = b^.^x^a^. Taking the logarithm (base 10) of each side of the equation, we performed a linear conversion and obtained the following equation: log (y) = log (b) + *a* log (x), where, x and y represent the below–and aboveground biomass, respectively. Log (b) represents the intercept of the regression equation between above–and belowground biomass, and *a* represents the slope of the regression equation, which is the relative growth index. The relative growth index suggests isometric growth if there is no significant difference between ‘a’ and ‘1.0,’ and it suggests allometric growth when ‘a’ is significantly larger or smaller than ‘1.0’ [32]. Standardized major axis estimation (SMA) was used to estimate the slope parameters and the intercept [33] using the SMATR Version 2.0 software (http://www.bio.mq.edu.au/ecology/SMATR). The Sigmaplot 11.0 software was used for plotting graphs. Differences in tree sizes (n = 100), the biomass, and BGB/AGB ratio (n = 23) of *L*. *leucocephala* between treatments were tested using an analysis of variance (ANOVA) and Tukey's test. Rejection level was set when α < 0.05 in all analyses. Analyses were conducted using SPSS 16.0 (IBM; Armonk, NY).

## Results

### Tree sizes

There was a significant difference in *L*. *leucocephala* growth among different plant stands (Table 1). *L*. *leucocephala* trees in monoculture (close spacing and wide spacing) were significantly taller and had significantly larger DBH and crown diameter than plants in mixed stands (M. W. *camaldulensis*, M. W. *citriodora*) (*p*<0.05). However, changing the plant spacing and mixture of tree species had no significant effect on growth.

**Table 1:** Descriptive statistics of the attributes measured on *L*. *leucocephala*.

Planting Patterns	Average diameter at breast height (DBH)/cm		Average height (H)/m		Average Crown diameter (CD)/ m	
	Mean	Range	Mean	Range	Mean	Range
Close spacing	11.3b	6.9–15.7	9.3b	7.7–12.1	5.6b	3.3–7.1
Wide spacing	11.8b	8.1–15.2	9.9b	6.3–12.9	5.9b	3.4–7.5
M. W. *camaldulensis*	9.1a	7.3–12.1	8.4a	5.4–11.9	4.7a	3.2–7.7
M. W. *citriodora*	8.7a	6.4–12.9	8.1a	5.0–10.6	4.4a	3.7–6.1

For each parameter, values with different letters are significantly different (*P* < 0.05).

### The biomass distribution and BGB/AGB ratio

Higher AGB, BGB, and TB as well as lower BGB/AGB ratios of *L*. *leucocephala* were observed in monoculture as compared to mixed plant stands (Table 2). AGB, BGB, TB, and BGB/AGB ratios showed a significant response to plant stands (monoculture or mixed plant stands) (*p*<0.05). Increasing the plant spacing significantly increased AGB and TB of *L*. *leucocephala* (*p*<0.05), and it had no significant effect on the biomass of BGB and BGB/AGB (*p*>0.05). Changing the mixture of tree species had no significant effect on the biomass distribution or BGB/AGB ratio of *L*. *leucocephala* (*p*>0.05).

**Table 2:** The above–and belowground biomass of *L*. *leucocephala* and the characteristics of the BGB/AGB values (n = 23).

Planting Patterns	Aboveground Biomass (AGB)/kg		Belowground Biomass (BGB)/kg		Total Biomass (TB)/kg		BGB/AGB Ratio (BGB:AGB)	
	Mean	Range	Mean	Range	Mean	Range	Mean	Range
Close spacing	30.5b	23.6–44.0	8.6b	6.7–12.1	39.1b	31.3–52.6	0.28a	0.21–0.40
Wide spacing	33.6c	22.1–48.3	8.7b	7.4–14.8	42.3c	30.7–62.0	0.26a	0.23–0.44
M. W. *camaldulensis*	19.4a	13.7–27.7	6.9a	4.5–10.7	26.3a	18.5–37.6	0.36b	0.27–0.43
M. W. *citriodora*	18.5a	11.3–23.5	7.2a	4.1–11.0	25.6a	16.0–33.1	0.39b	0.28–0.46

For each parameter, values with different letters are significantly different (*P* < 0.05).

### Distribution characteristics of aboveground biomass

**Fig 1:**
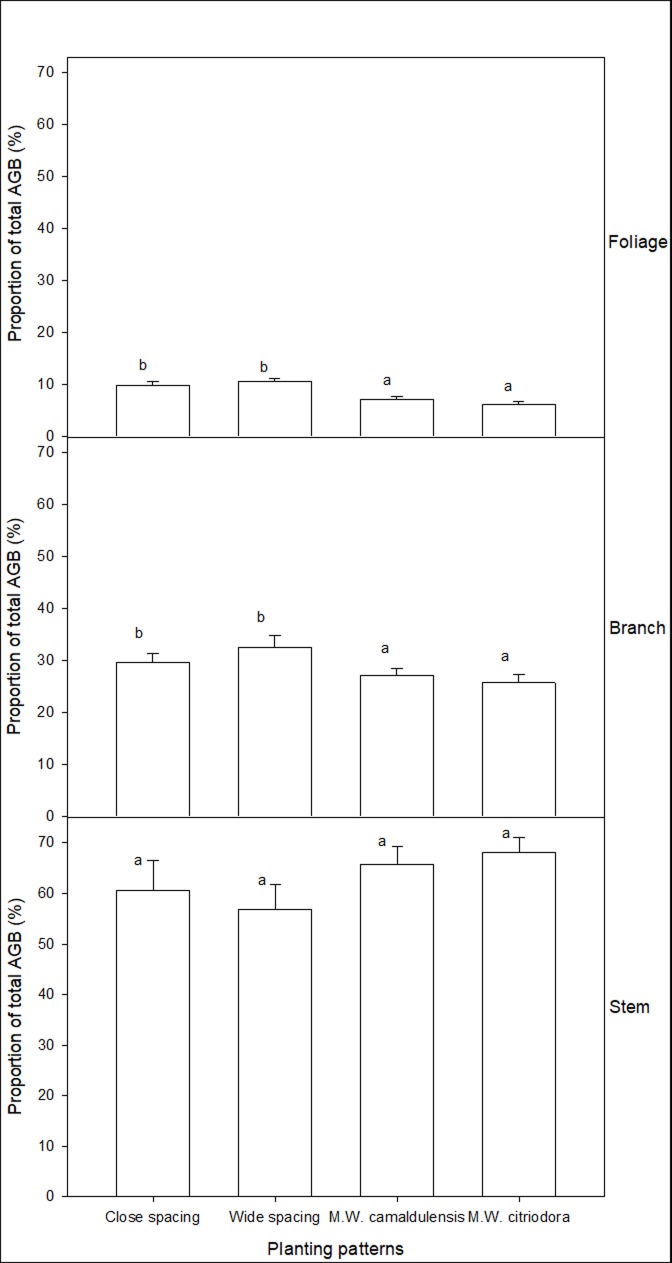
The characteristics of aboveground biomass distribution of *L*. *leucocephala*. Close spacing represent 100% *L*. *leucocephala* at spacing of 1.5 m×3.0 m; wide spacing represent 100% *L*. *leucocephala* at spacing of 3.0 m×3.0 m; M. W. *camaldulensis* represent 50% *L*. *leucocephala* + 50% *E*. *camaldulensis* at 3.0 m×3.0 m spacing; M. W. *citriodora* represent 50% *L*. *leucocephala* + 50% *E*. *citriodora* at 3.0 m×3.0 m spacing. Different letters between bars indicate significant differences (*p*<0.05).

The AGB of *L*. *leucocephala* was mainly concentrated in the stem, with a lowest overall proportion of AGB of 55.9% (in Wide spacing of the monoculture) and a highest proportion of 68.1% (in M. W. *citriodora* of the mixed plant stands), and no significant difference was found in stem biomass between different plant stands (*p*>0.05) (Fig 1). Higher branch and foliage biomasses were observed in monoculture than in mixed plant stands. Changing the plant spacing and mixture of tree species had no significant effect on the distribution characteristics of AGB.

### Distribution characteristics of belowground biomass

**Fig 2:**
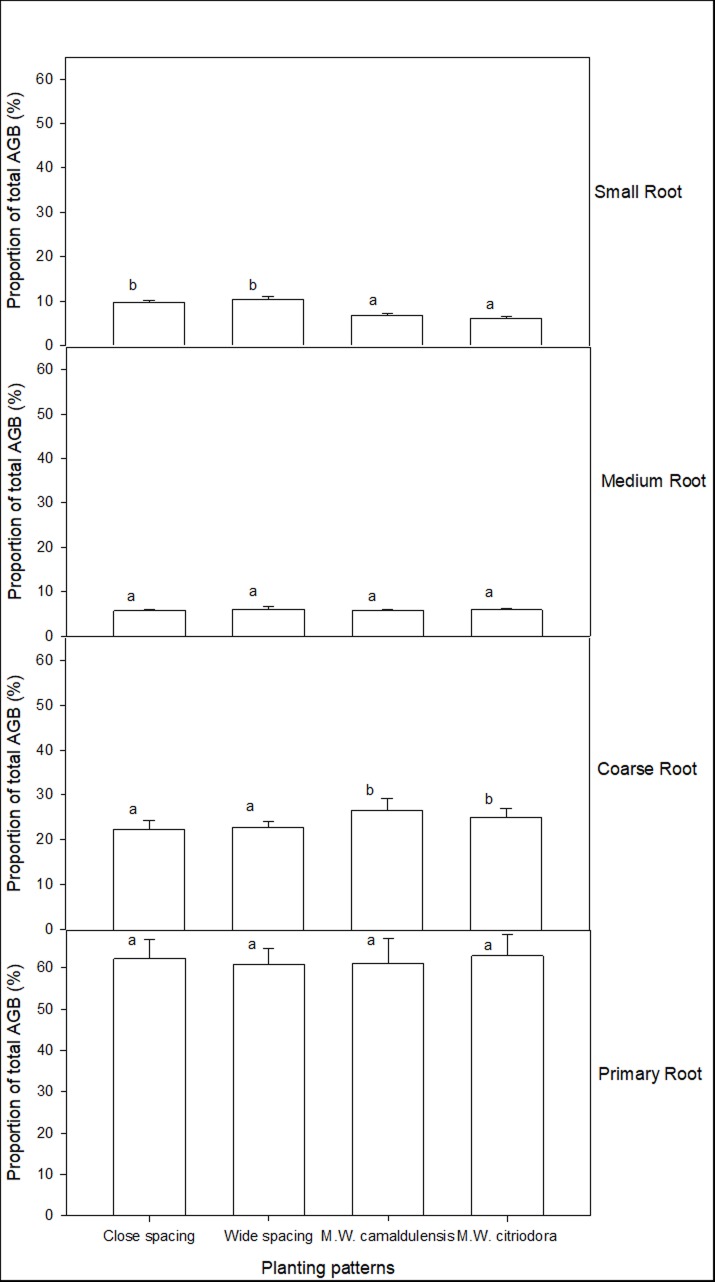
The characteristics of belowground biomass distribution of *L*. *leucocephala*. Close spacing represent 100% *L*. *leucocephala* at spacing of 1.5 m×3.0 m; wide spacing represent 100% *L*. *leucocephala* at spacing of 3.0 m×3.0 m; M. W. *camaldulensis* represent 50% *L*. *leucocephala* + 50% *E*. *camaldulensis* at 3.0 m×3.0 m spacing; M. W. *citriodora* represent 50% *L*. *leucocephala* + 50% *E*. *citriodora* at 3.0 m×3.0 m spacing. Different letters between bars indicate significant differences (*p*<0.05).

The BGB of *L*. *leucocephala* was mainly concentrated in the primary root, with the lowest overall proportion of BGB at 60.4% (in Wide spacing of the monoculture) and the highest proportion at 63.2% (in M. W. *citriodora* of the mixed plant stands) (Fig 2). No significant difference was found in the primary root biomass between different plant stands (*p*>0.05). Significantly higher coarse root biomass and lower small root biomass were observed in mixed plant stands when compared to monoculture (*p*<0.05). Changing the plant spacing and mixture of tree species had no significant effect on the distribution characteristics of BGB.

**Fig 3:**
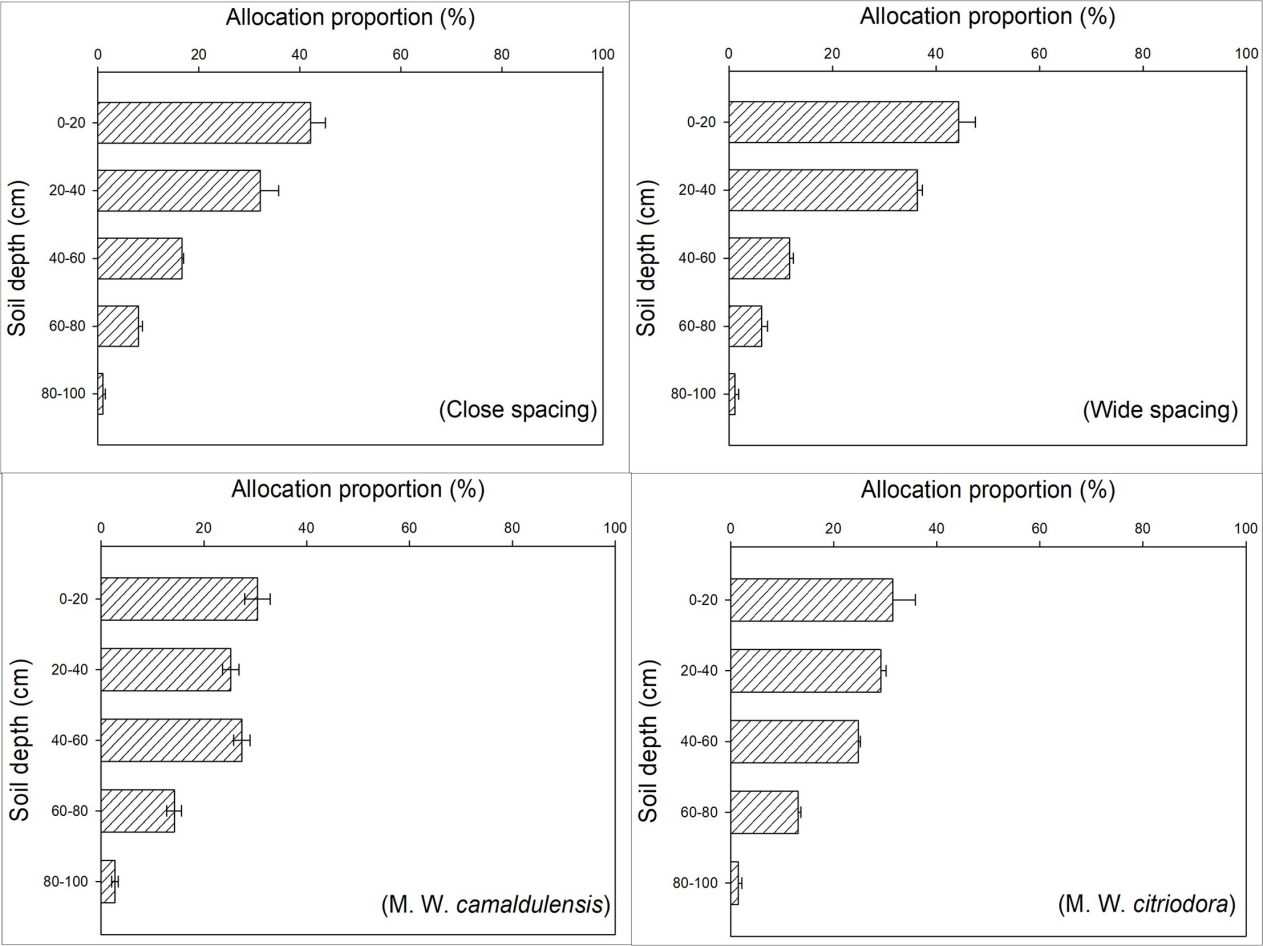
The vertical distribution of belowground biomass of *L*. *leucocephala* in different planting patterns. Close spacing represent 100% *L*. *leucocephala* at spacing of 1.5 m×3.0 m; wide spacing represent 100% *L*. *leucocephala* at spacing of 3.0 m×3.0 m; M. W. *camaldulensis* represent 50% *L*. *leucocephala* + 50% *E*. *camaldulensis* at 3.0 m×3.0 m spacing; M. W. *citriodora* represent 50% *L*. *leucocephala* + 50% *E*. *citriodora* at 3.0 m×3.0 m spacing.

The BGB of *L*. *leucocephala* decreased gradually with depth, and it was mostly concentrated in the soil surface between 0–40 cm (Fig 3). Statistical comparison of means showed that BGB within the top 0–40 cm of the surface soil in monoculture (74.30% in Close spacing; 80.79% in Wide spacing) was significantly higher than that in the mixed plant stands (55.66% in M. W. *camaldulensis*; 60.59% in M. W. *citriodora*) (*p*<0.05).

We analyzed the vertical distribution of root systems using RSDM(*Y* = 1-*β*^*d*^). The root system distribution value *β* of *L*. *leucocephala* was 0.933 (R^2^ = 0.823, *p*<0.01) in Close spacing, 0.912 (R^2^ = 0.907, *p*<0.01) in Wide spacing, 0.951 (R^2^ = 0.941, *p*<0.01) in M. W. *camaldulensis*, and 0.942 (R^2^ = 0.769, *p*<0.01) in M. W. *citriodora*. Therefore, the root system biomass concentrated in the surface soil followed the pattern: Wide spacing > Close spacing > M. W. *citriodora* > M. W. *camaldulensis*.

### The relationship between above–and belowground biomass

**Fig 4:**
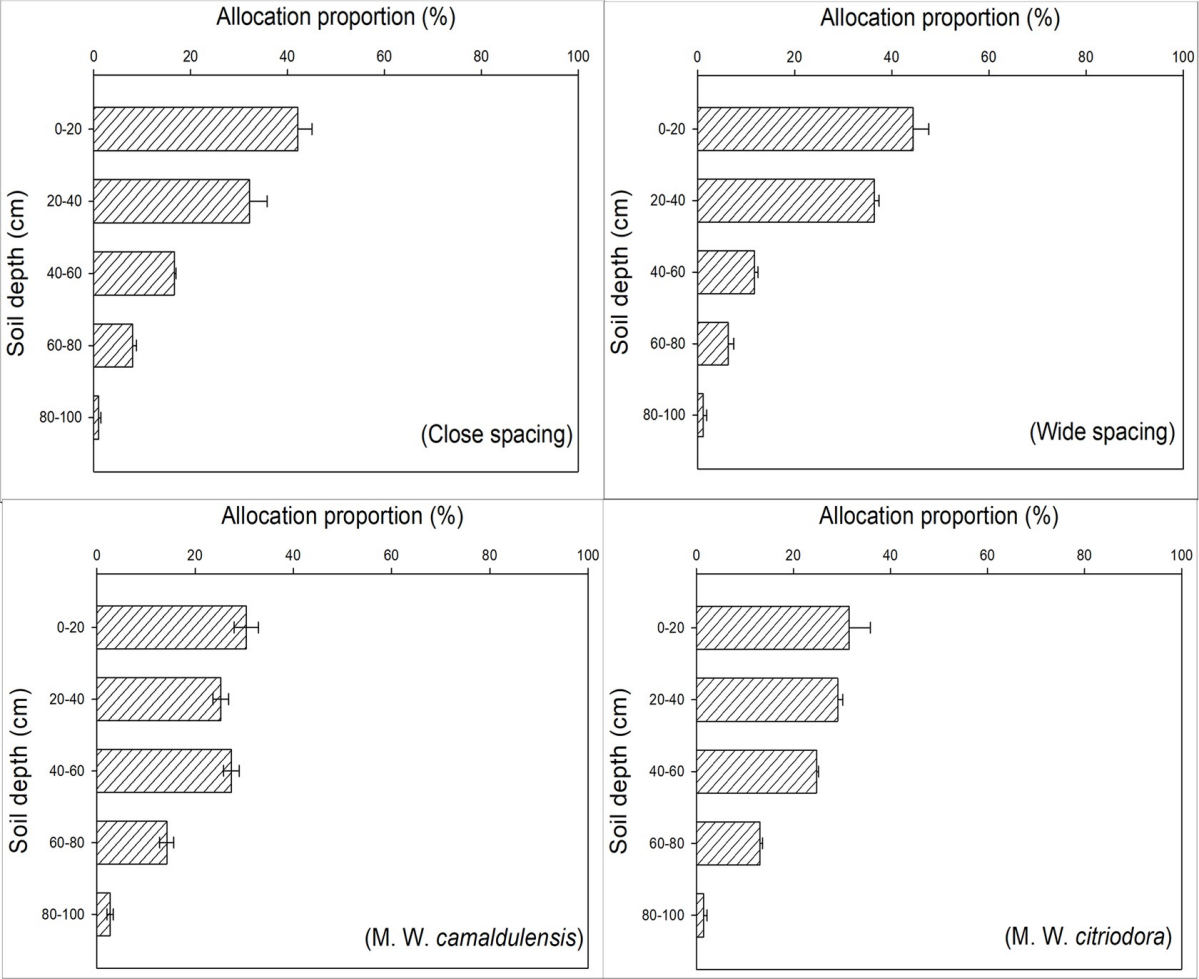
The relationships between AGB and BGB of *L*. *leucocephala* in different plant stands. Close spacing represent 100% *L*. *leucocephala* at spacing of 1.5 m×3.0 m; wide spacing represent 100% *L*. *leucocephala* at spacing of 3.0 m×3.0 m; M. W. *camaldulensis* represent 50% *L*. *leucocephala* + 50% *E*. *camaldulensis* at 3.0 m×3.0 m spacing; M. W. *citriodora* represent 50% *L*. *leucocephala* + 50% *E*. *citriodora* at 3.0 m×3.0 m spacing.

*Log*BGB of *L*. *leucocephala* in different plant stands was closely correlated with *Log*AGB, and a strong linear relationship was found between them (Fig 4). Standardized major axis estimation (SMA) analysis showed that the slope of the linear regression for *Log*BGB and *Log*AGB was 1.117 for Close spacing (95% Confidential Interval (CI): 0.862–1.449), 1.002 for Wide spacing (95% CI: 0.852–1.179), 0.795 for M. W. *camaldulensis* (95% CI: 0.648–0.974), and 0.793 for M. W. *citriodora* (95% CI: 0.661–0.951) (Table 3). Among these, the fitting slopes of *L*. *leucocephala* grown in monoculture (close spacing and wide spacing) showed no significant difference from 1.0 (*p* (H0: slope) > 0.05), indicating isometric growth of AGB and BGB. The fitting slopes of *L*. *leucocephala* grown in mixed plant stands (M. W. *camaldulensis* and M. W. *citriodora*) were significantly smaller than 1.0 (*p* (H0: slope) <0.05). This indicated allometric growth of AGB and BGB, and illustrates that BGB increased faster than AGB.

**Table 3:** Standardized major axis estimation (SMA) analysis of the above–and belowground biomass distribution.

Planting Patterns	Sample Size	R^2^	Slope (95%CI)	Intercept	*p*(H0:slope = 1)
Close spacing	23	0.665	1.117(0.862–1.449)	0.437	0.389
wide sapcing	23	0.870	1.002(0.852–1.179)	0.575	0.977
M. W. *camaldulensis*	23	0.795	0.795(0.648–0.974)	0.623	0.029*
M. W. *citriodora*	23	0.837	0.793(0.661–0.951)	0.586	0.014*

*denotes significant difference (*P*<0.05)

## Discussion

In the present study, almost all metrics of *L*. *leucocephala* tree size were greater in monoculture (Tables 1 and 2). All treatments showed a significant response to planting stand (monoculture or mixed plant stands) (*p*<0.05), reflecting increased competition for *L*. *leucocephala* in the mixed plant stands. However, changing the plant spacing and mixture of tree species had no significant effect on most growth metrics and BGB/AGB ratio. This indicated that mixed plantations with *Eucalyptus* significantly influenced tree sizes and biomass distribution of *L*. *leucocephala*,but spacing did not affect these characteristics.

The results also suggested that interspecific competition was higher than intraspecific competition for *L*. *leucocephala*. Similarly, mixtures that contain nitrogen–fixing species, such as ‘*E*. *globules* + *Acacia mearnsii*,’ ‘*E*. *saligna* + *Facaltaria moluccana*,’ and ‘*Eucalyptus grandis* + *Acacia mangium*,’ were found to have significant effects on plantation yields [5, 21–22]. However, these studies showed that the tree sizes of nitrogen–fixing trees increased in the mixed plant stands, which contrasted with the results of the present study. The differences in results can be explained by the different age of plant stands and the different growth environment. In the studies by Laclau et al. [5] and Bauhus et al. [21], investigations were respectively carried out 30 months and 6.5 years after planting, when there was less intense competition in the plantation. With continued tree growth, competition within the forest would become increasingly intense, and the situation might change significantly. On the other hand, Binkley et al. [22] established their experiments at 480 m elevation on the northeast coast of Hawaii, where the average annual rainfall was approximately 4000 mm. Under such conditions, nitrogen fixing trees might have adapted to use the higher water supply and soil fertility via developing stronger stems and root systems, and the complementarities between nitrogen fixing trees and non–nitrogen fixing trees were therefore greater than their competition for resources in the mixed plantations.

Furthermore, the non–nitrogen fixing trees of *Eucalyptus* in the mixed plantations was a fast growing plant with growth rates that routinely exceeded 35 m^3^ ha^-1^ year^-1^ [34–35]. It could also be seen from our survey data that the *Eucalyptus* had larger DBH values (2.0–2.2 times), heights (1.3–1.7 times), and crown diameters than *L*. *leucocephala* in the mixed plantations (see the Materials and methods section and Table 1). However, there is an evident negative impact of *Eucalyptus* on soil water availability for other species [36–38]. When *Eucalyptus* is grown, the land of mixed plantations would become more water–impoverished. As a result, the competition for water between *Eucalyptus* and *L*. *leucocephala* is more intense, and the growth of *L*. *leucocephala* can be restrained.

The root system is the most important organ of plant nutrition. Reduction in tree sizes of *L*. *leucocephala* in the mixed planting stands might be determined by the structure and interactions of the root systems. In the present study, results suggested that the top 0–40 cm of the surface soil was very important for *L*. *leucocephala* for absorbing nutrients and water as well as the exchange of materials with the external environment. Furthermore, BGB within the top 0–40 cm of the surface soil in monoculture was significantly higher than that in the mixed plant stands (*p*<0.05) (Fig 3). The results of RSDM suggested that mixtures had lower *β–*values than monoculture. This showed that *L*. *leucocephala* in the mixed plant stands had deeper root systems than in monoculture. Simultaneously, when planted with *Eucalyptus*, *L*. *leucocephala* significantly reduced the biomass allocation to leaves and small roots as well as increased coarse root biomass (*p*<0.05) (Figs 2 and 3). This indicated that more competition was found in the roots of *L*. *leucocephala* in the mixed plant stands and the coarse root competition was particularly strong. It suggested that the water absorption of *L*. *leucocephala* in mixed plant stands was more restricted than in monoculture. This might be related to the suppression of *L*. *leucocephala* growth by tall Eucalyptus and the higher water consumption characteristics of *Eucalyptus* [37–38]. Tang et al. [39] indicated that there were no significant interactions between the roots of *L*. *leucocephala* and *Eucalyptus* 10 years after establishment of the mixed plant stands in the valley–type savanna. This was due to the fact that rocky soil inhibited root growth. However, the situation might be changed after 17 years of trees growth. This was similar to some studies in other regions [40–41]. As plants grow, fierce competition between the roots of *Eucalyptus* and *L*. *leucocephala* were found in the mixed plant stands, although *L*. *leucocephala* could provide nitrogen via fixation.

Isometric allocation hypotheses and allometric allocation hypotheses for the relationship between AGB and BGB of the individual plants have been widely evaluated based on field observations. Many studies on the biomass allocation of trees grown in good conditions have supported the existence of isometric growth between AGB and BGB [3, 14, 42]. However, in harsh ecosystems, the allometric allocation hypothesis between AGB and BGB has often been confirmed. Plants would allocate more biomass to competing organs, thereby increasing the absorption and utilization of limited resources [10, 43–44]. In the present study, the relationship between AGB and BGB of *L*. *leucocephala* in monoculture with different spacing exhibited isometric growth, which supported the isometric allocation hypothesis. The mixed plant stands showed allometric growth, which supported the allometric allocation hypothesis. Furthermore, BGB increased faster than AGB (Table 3). This showed that the competition for available water content in the mixed plant stands was more intense. This was consistent with the results of the previous analysis on tree size of *L*. *leucocephala* in different planting lands. With increasing of water stress, plants would allocate more biomass to the root system and less to the AGB [8–10]. Therefore, biomass allocation in monoculture and mixed plant stands might support the different allocation hypothesis. Although the allocation relationship between BGB and AGB is not equivalent to the BGB/AGB ratio, they become comparable in different plant stands. In the present study, values of BGB/AGB ratio in mixed plant stands were significantly increased (Table 2). Generally, BGB to AGB ratios have a tendency to increase in drier, harsher conditions [8, 10]. These results suggested that there was a significant change in the proportion of AGB and BGB allocation for *L*. *leucocephala* when planted with *Eucalyptus*, and more biomass was allocated to the root system. In addition, these values were higher than the IPCC [45] default value of 0.25 for hardwood species and thus might significantly increase the estimate of BGB of *L*. *leucocephala* in the dry and hot environment, especially for mixed plantations.

## Conclusion

Our results suggested that mixed plantations with *Eucalyptus* significantly decreased almost all growth metrics of *L*. *leucocephala*, including DBH, height, average crown diameter, AGB, BGB and TB, while it significantly increased BGB/AGB ratios. Increasing the plant spacing only significantly increased AGB and TB, and had no significant effect on the other metrics. Compared with in monoculture, *L*. *leucocephala* in mixed plantations with *Eucalyptus* significantly reduced the biomass allocation to leaves and small roots, and the plants increased the allocation to coarse root biomass. This study also showed that the correlation between AGB and BGB of *L*. *leucocephala* in all plant stands was consistent with the model of allometric growth. However, the correlation was not exactly the same. It showed isometric growth in monoculture, and allometric growth in mixed plant stands.

## Supporting information

S1 Dataset (XLSX)
